# Enhanced physical properties of γ-Al_2_O_3_–rGO hybrids prepared by solvothermal and hot-press processing†

**DOI:** 10.1039/c8ra00095f

**Published:** 2018-02-22

**Authors:** Mujtaba Ikram, Zhuchen Tao, Jianglin Ye, Hafiz Adil Qayyum, Xuemei Sun, Jin Xu

**Affiliations:** Key Laboratory of Materials for Energy Conversion, Chinese Academy of Sciences, Department of Materials Science and Engineering, University of Science and Technology of China Hefei Anhui 230026 P. R. China mujtaba@mail.ustc.edu.cn; Physics Department, King Fahd University of Petroleum and Minerals Dhahran 31261 Saudi Arabia

## Abstract

In this study, a solvothermal method was employed for the first time to fabricate hybrids composed of cross-linked γ-Al_2_O_3_ nanorods and reduced graphite oxide (rGO) platelets. After calcination and hot-press processing, monoliths of Al_2_O_3_–rGO hybrids were obtained with improved physical properties. It was found that the oxygen-containing groups on graphene oxide were beneficial for the adsorption of aluminum isopropoxide, leading to a uniform dispersion of rGO with Al_2_O_3_, which was obtained by hydrolysis of aluminum isopropoxide during the solvothermal reaction. The hybrid, which was subsequently calcinated for 3 h showed electrical conductivity of 6.7 × 10^1^ S m^−1^ together with 90% higher mechanical tensile strength and 80% higher thermal conductivity as compared to the bare Al_2_O_3_. In addition, the dielectric constant of the hybrid was 12 times higher than that of the bare Al_2_O_3_. In this study, the highest values of electrical conductivity (8.2 × 10^1^ S m^−1^), thermal conductivity (2.53 W m^−1^ K^−1^), dielectric constant (10^4^) and Young's modulus (3.7 GPa) were obtained for the alumina–rGO hybrid calcinated for 1 h. XRD characterization showed that an increase in calcination temperature and further hot-press processing at 900 °C led to enhanced crystallinity in the γ-Al_2_O_3_ nanorods in the hybrid, resulting in enhanced physical properties in the hybrids.

## Abstract 2

After calcination and hot-press processing, monoliths of γ-Al_2_O_3_–rGO hybrids are obtained with improved physical properties.

## Graphical abstract

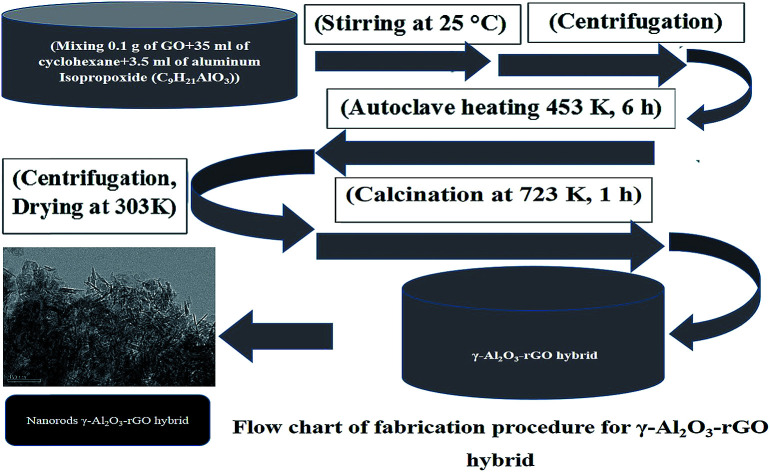

## 1. Introduction

Due to its high thermal conductivity (5000 W m^−1^ K^−1^), high electrical conductivity (10^6^ S m^−1^), high Young's modulus (1 TPa) and high intrinsic strength (130 GPa),^1,2^ graphene is considered as an ideal candidate to improve the electrical, mechanical and thermal properties of metals, polymers and ceramics.^3^ Among the numerous types of graphene materials, graphite oxide derived graphene plays a significant role in enhancing the properties of hybrids because of its tunable surface functionalization and the potential for large-scale production.^4–7^ For example, reduced graphite oxide (rGO)–polystyrene composites with a low threshold content of rGO of ∼0.1 volume percent have shown greatly improved electrical conductivity (∼0.1 S m^−1^) due to the good dispersion of rGO in the composites.^8^ In inorganic hybrids, rGO has been used for the deposition of Co_3_O_4_ particles for enhanced catalytic effects for the decomposition of ammonium perchlorate due to the integrated properties of the GO and Co_3_O_4_ nanoparticle-components.^9^ In another study, rGO was used to enhance the toughness of bulk silicon nitride by up to ∼235% (from ∼2.8 to ∼6.6 MPa m^1/2^), which may be used for high performance structural applications.^10^

Ceramics usually have a brittle attribute with low strength.^11^ Among the numerous ceramics, alumina is one of the widely used structural ceramic due to its good thermal conductivity and shape capability.^12^ Alumina has applications in the field of dental implants, high speed cutting tools, chemical insulators, electrical insulators, and wear resistance coatings.^13,14^ To improve its mechanical properties, carbon nanotubes have been used to enhance the fracture toughness (by 94%), hardness (by 13%) and flexural strength (by 6.4%) of alumina.^15^ Ball-milled alumina/zirconia/graphene composites have been investigated with 40% enhancement in fracture toughness on addition of graphene platelets.^16^ In another study, alumina–rGO core–shell nanocomposites were fabricated using the dry sol gel method, which indicated that the BET surface area of rGO is essential to enhance the surface charge properties of hybrids.^17^ In another study, alumina–graphene composite films were reported with low optical gap (1.53 eV).^18^ Alumina–rGO nanocomposites obtained *via in situ* deposition showed a unique morphology of alumina nanoparticles on rGO with a BET surface area of 242.4 m^2^ g^−1^ and low porosity.^19^ An alumina/rGO/poly(ethylenimine) composite was used to capture carbon dioxide from flue gas.^20^ In a microwave preparation of alumina–rGO composites, the grain size of the alumina matrix was reduced to 180 nm as compared to 475 nm grain size obtained from the conventional sintering process, leading to an increase in the Young's modulus of 180 GPa from 148 GPa under the same measurement conditions.^21^ However, in most of the previous reports on alumina–rGO, there is no complete systematic study on the physical properties of Al_2_O_3_–rGO such as investigation of dielectric, mechanical, electrical, and thermal properties.

In this study, we report the preparation of hybrids consisting of γ-Al_2_O_3_ nanorods and rGO by a solvothermal method. For the first time, the solvothermal method was used to form hybrids composed of cross-linked γ-Al_2_O_3_ nanorods and reduced graphite oxide (rGO) platelets. Upon further hot pressing, hybrid monoliths were obtained to systematically study the enhanced physical properties of the hybrids. It is found that with the 3 h-calcinated hybrid, the Al_2_O_3_–rGO monoliths show enhanced electrical conductivity (ranging from 5.1 × 10^−10^ S m^−1^ to 6.7 × 10^1^ S m^−1^), mechanical tensile strength (90%), thermal conductivity (80%) and a much higher dielectric constant (12 times) than the bare Al_2_O_3_. In this study, the highest values of electrical conductivity (8.2 × 10^1^ S m^−1^), thermal conductivity (2.53 W m^−1^ K^−1^), dielectric constant (10^4^) and Young's modulus (3.7 GPa) are determined for the alumina–rGO hybrid calcinated for 1 h. It was found that the oxygen-containing functional groups on GO were beneficial for the adsorption of aluminum isopropoxide, leading to a uniform dispersion of rGO with Al_2_O_3_, which was formed due to the hydrolysis of aluminum isopropoxide during the solvothermal process. The study of the aspect ratio of the nanorods indicates that the elongated γ-Al_2_O_3_ nanorods are the major cause for the improvement in mechanical properties. Furthermore, for the Al_2_O_3_–rGO monoliths, the effect of calcination temperature on the length, diameter and aspect ratio of the alumina nanorods is discussed.

## 2. Experimental

### 2.1 Preparation of γ-Al_2_O_3_–rGO hybrid powder

In brief, the preparation of the γ-Al_2_O_3_–rGO hybrids was carried out by mixing GO with cyclohexane and aluminum isopropoxide (C_9_H_21_AlO_3_), followed by a solvothermal reaction. For the preparation, 0.1 g of GO was first dispersed in 35 mL cyclohexane, following which 3.5 mL of aluminum isopropoxide (C_9_H_21_AlO_3_) was added dropwise. The mixture was then stirred at a speed of 1000 rpm at room temperature for several days until the GO powder was homogeneously dispersed, following which the color of the suspension remained constant. Centrifugation was used to separate the products, which were then washed several times with cyclohexane. The solid samples obtained are denoted as Al(O)_*x*_/GO. Al(O)_*x*_/GO was dispersed in 50 mL cyclohexane and then transferred to a 100 mL Teflon-lined stainless-steel autoclave for hydrothermal treatment. After the reaction was carried out at 453 K for 6 h, the resultant sample was again centrifuged and dried at 303 K, which is denoted as Al(O)_*x*_/rGO. Al(O)_*x*_/rGO was then calcinated at 723 K for 1, 2, and 3 h to form γ-Al_2_O_3_/rGO hybrids. Calcination was performed under a limited supply of air. To control the calcination process, it was carried out in a quartz tubular furnace with open ends that allowed calcination under a limited supply of air; the furnace was heated to the desired temperature of 723 K for calcination times of 1, 2 and 3 h. Initially, a heating rate of 15 °C min^−1^ was used to increase the temperature. The calcination treatment resulted in the formation of free standing γ-Al_2_O_3_ nanorods and the reduction of GO to rGO. γ-Al_2_O_3_/rGO hybrid powder samples consisting of 16.707, 12.830 and 7.705 wt% of rGO were obtained using the same method for studying the physical properties. The same procedure was also used to form pure γ-Al_2_O_3_ without the addition of GO. For analysis of the effect of calcination temperature on crystallinity, the calcination temperature was set as 500 K, 600 K, 650 K, 700 K, 750 K and 800 K, for a processing time of 1 h. For analysis of the effect of calcination temperature and time on the nanorod structure, the calcination time was set as 1, 2, 3, 4 and 5 h for the temperatures of 723 K, 823 K and 923 K.

### 2.2 Hot press processing of γ-Al_2_O_3_/rGO hybrids

Hot pressing of the γ-Al_2_O_3_/rGO hybrid powder was performed in a vacuum furnace (model number OTF-1200X-VHP4). The furnace was integrated with an electric hydraulic press to compress the samples in a graphite pressing die. The temperature was set to increase from room temperature at a heating rate of 10 °C min^−1^ up to 900 °C and then, this temperature was maintained for 60 minutes. When the temperature reached 80% of the set temperature, pressure of 25–30 MPa was applied to the hybrids.

### 2.3 Characterization

Scanning electron microscopy (SEM) and energy dispersive X-ray spectroscopy (EDS) were performed using JSM-6700F (operated at 10 kV). The specific Brunauer–Emmett–Teller (BET) surface area was determined using a Micromeritics instrument (TriStar II 3020). The structure of the hybrids was characterized *via* X-ray diffraction (XRD) using a powder diffractometer (Smart Lab, Rigaku, Japan) and Cu Kα radiation (*λ* = 0.15414 nm). Thermogravimetric analysis (TGA) was carried out using a TG analyzer (DTG-60H, Shimadzu, Japan) under flowing air from room temperature to 800 °C with a step of 10 °C min^−1^. Transmission electron microscopy (TEM) images and selected area electron diffraction (SAED) images were taken on a TEM (JEM-2100F, 200 kV, JEOL, Japan). Raman spectra were recorded using a Raman microscope (inVia, Renishaw, 532 nm laser). Mechanical compression was measured using an MTS 809 Axial Torsional Test System, in which the compression rate was 0.22 mm min^−1^. Mechanical tensile stress strain curves were obtained with a ramp force of 0.5 N min^−1^ up to 18 N using a dynamic mechanical analyzer (DMA, Q800, TA Instrument). Electrical conductivity was measured using the 4-probe method with a digital duel measurement system (Gwinstek, GDM-8261A). Dielectric property was measured using an LCR Meter (Tonghui, TH2811DN). Thermal conductivity was measured using a laser flash thermal conductivity instrument with an energy pulse of up to 18 J per pulse (Netzsch, LFA457). Statistical analysis of the average lengths and diameters of the nanorods was conducted using the Gatan Digital Micrograph 3.9 software. Each physical measurement was carried out 10 times and the average values are shown with error bars in the following sections.

## 3. Results and discussion

**Fig. 1:**
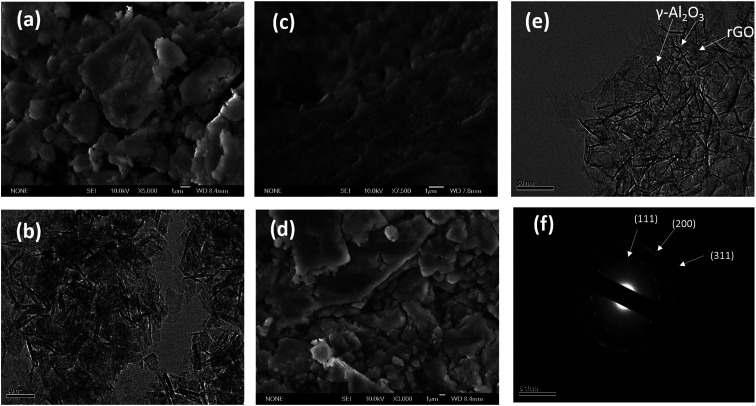
(a) SEM and (b) TEM images of pure γ-Al_2_O_3_. SEM images of Al(O)_*x*_/GO (c) before calcination at an autoclave heating temperature of 453 K for 6 h and (d) after calcination at 723 K for 2 h. (e) TEM image of γ-Al_2_O_3_–rGO after calcination at 723 K for 2 h, and (f) SAED pattern of the γ-Al_2_O_3_–rGO hybrid at 723 K for 2 h.

The TGA curves (shown in Fig. S1a†) of the γ-Al_2_O_3_–rGO powder samples show that different calcination times led to different concentrations of rGO in the hybrids. The TGAs curves of all the hybrids show a stable weight loss between 400 °C and 600 °C, which is ascribed to the removal of all the carbon-related materials,^20,22^ and other impurities (if any) after heating to 800 °C in air atmosphere. For the sample with a 3 h-calcination time, the calculated loss was 7.705 wt%. For the sample with a 2 h-calcination time, the calculated loss was 12.830 wt%. For the sample with a 1 h-calcination time, the calculated loss was 16.707 wt%. The SEM image [Fig. 1a] of bare Al_2_O_3_ exhibits a particle-like morphology. The size of the particles range from 500 nm to a few micrometers. The TEM image [Fig. 1b] shows elongated nanocrystals or nanorods of bare Al_2_O_3_. The sample before calcination but after autoclave heating is denoted as Al(O)_*x*_/rGO.^22^ The SEM image of Al(O)_*x*_/rGO after heating in an autoclave at a temperature of 453 K for 6 h but before calcination is shown in Fig. 1c. With rGO in the hybrids, the sample powder exhibited a color change after calcination as shown in the optical images in Fig. S1b and c.† Even after calcination at 723 K for 2 h, the SEM image (Fig. 1d) shows the same particle-like morphology, but the size of the particles range from 1 micrometer to a few micrometers. The TEM image of the γ-Al_2_O_3_–rGO hybrids after calcination at 723 K for 2 h is shown in Fig. 1e, where elongated and fine nanorods of γ-Al_2_O_3_ with an rGO layer in the hybrids are observed. The TEM image shown in Fig. 1e indicates the presence of a very thin rGO layer, which acts as a continuum matrix in the hybrids. The presence of rGO can also be confirmed by closely observing Fig. 1e. In this figure, the low-contrast features are actually edges or small portions of the graphene sheet (Fig. 1e), on which γ-Al_2_O_3_ is uniformly distributed in dense concentrations. This is a key requirement for good properties in hybrid γ-Al_2_O_3_–rGO after calcination. Further, the selected area electron diffraction pattern presented in Fig. 1f shows the interplanar spacings of *D* = 0.175 nm and *D* = 0.151 nm, which correspond to the (200) and (111) planes of γ-Al_2_O_3_, respectively.

**Fig. 2:**
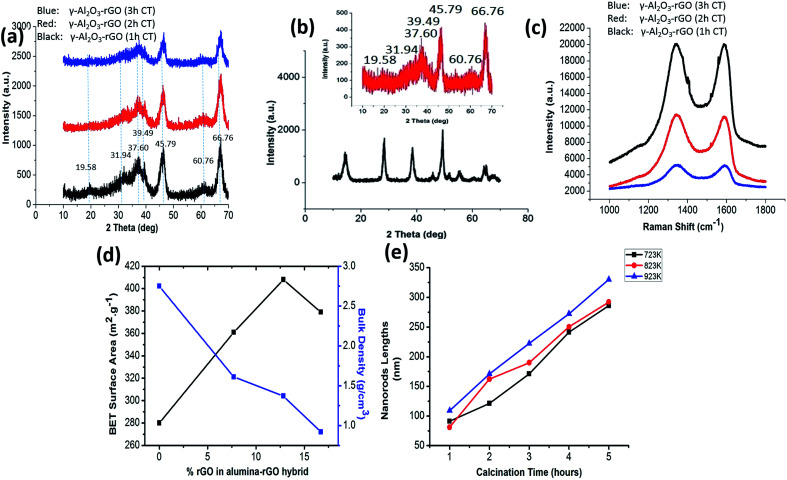
(a) XRD of γ-Al_2_O_3_–rGO (1, 2 and 3 h calcination time), (b) XRD pattern of γ-Al_2_O_3_–rGO before calcination and inset is the XRD pattern of pure γ-Al_2_O_3_, fabricated at 723 K, (c) Raman spectra of γ-Al_2_O_3_–rGO (1, 2 and 3 h calcination time), (d) BET surface area and bulk density of γ-Al_2_O_3_–rGO as function of % rGO in the hybrid and (e) average nanorod length as a function of calcination time.

The successful fabrication of the γ-Al_2_O_3_ phase was confirmed from the XRD results as shown in Fig. 2a and b. The inset of Fig. 2b shows the XRD pattern of the sample without GO (γ-Al_2_O_3_). In the XRD spectra of all three samples with calcination times of 1, 2, and 3 h, the characteristic peaks of γ-Al_2_O_3_ are evident (consistent with JCPDS card no. 10-0425).^23^ Further, sharp peaks with relatively broad features are observed in the XRD spectra of γ-Al_2_O_3_ and the rGO hybrids after calcination [Fig. 2a and in the inset of Fig. 2b (for pure γ-Al_2_O_3_)]. This suggests that γ-Al_2_O_3_ has a nanocrystalline structure with nanorod morphology, which is also quite evident from the TEM images (Fig. 1e). The peak for rGO usually appears at 24.50° in the hybrids;^20^ however, this peak was not present in the XRD spectra of the samples after autoclave heating and calcination treatment. In the XRD spectra of the alumina–rGO hybrids, the absence of the carbon peak may be due to uniform mixture of rGO in alumina. In all cases after calcination for 1, 2 and 3 h, the characteristic peak of GO, which usually appears at 10.28°,^19,20^ is also absent in the hybrids [Fig. 2a]. The total changes in the peaks in the XRD pattern before and after calcination indicate that the effects of the hydrothermal and calcination treatments on the hybrids are beneficial for the formation of the γ-Al_2_O_3_ nanostructures and the reduction of GO.

Raman spectroscopy was conducted on the hybrids to confirm the presence of carbon as shown in Fig. 2c. The Raman spectra of the samples indicate that rGO is present in the hybrids calcinated for 1, 2 and 3 h. For the samples calcinated for different times (1, 2, and 3 h), the Raman intensity decreases with calcination time, which is consistent with the TGA results. The lowest intensity is found in the sample calcinated for 3 h (Fig. 2c). From Raman spectra, it is clear that the maximum intensity of the carbon peak (G peak) is observed in the sample calcinated for 1 h, which is consistent with the TGA results. However, the Raman spectra do not show any characteristic peaks for γ-Al_2_O_3_. The G-band value is different in the Raman spectra of all the γ-Al_2_O_3_ and rGO hybrids as compared to that of pristine graphene. This reveals the presence of prominent electronic interactions between γ-Al_2_O_3_ and rGO in the hybrids.^19,20^

To gain further insight into the structural properties, the Brunauer–Emmett–Teller (BET) surface areas of the samples were determined, which are shown in Fig. 2d. The N_2_ adsorption–desorption and pore size distribution curves for 1 h-calcinated γ-Al_2_O_3_ and 1 h-calcinated γ-Al_2_O_3_–rGO hybrids are shown in Fig. S2(a–d).† The average pore size of γ-Al_2_O_3_–rGO calcinated for 1 h is 12 nm, while that for γ-Al_2_O_3_ calcinated for 1 h, it is 17 nm. The presence of rGO helps to increase the surface area and results in a relatively smaller pore diameter compared to that of bare γ-Al_2_O_3_. The smaller pore diameter in the alumina–rGO hybrid compared to γ-Al_2_O_3_ indicates the formation of very well-defined mesopores, which may be caused by the entanglement of the thin nanorods of γ-Al_2_O_3_ on the rGO layer. Mesoporosity is also confirmed from the N_2_ adsorption–desorption and pore size distribution curves (Fig. S2†). The nanohybrids of γ-Al_2_O_3_–rGO calcinated for 3, 2, and 1 h have BET surface areas of 361, 408, and 379 m^2^ g^−1^, respectively. For bare γ-Al_2_O_3_, its BET surface area is 280 m^2^ g^−1^. The bulk densities of γ-Al_2_O_3_–rGO calcinated for 3, 2, and 1 h are 1.61, 1.37, and 0.92 g cm^−3^, respectively. For bare γ-Al_2_O_3_, its bulk density is 2.75 g cm^−3^. Clearly, the presence of rGO led to high BET surface area values. A comparison with previous reports on the BET surface area and density of γ-Al_2_O_3_ and rGO hybrids and γ-Al_2_O_3_ is summarized in Table S1.†^17,21^ The controlled, compact and elongated oriented entanglement of γ-Al_2_O_3_ nanorods on the rGO layer resulted in a higher surface area and lower density in γ-Al_2_O_3_–rGO as compared to bare γ-Al_2_O_3_. Thus, the presence of rGO in hybrids can increase their BET surface area, pore volume and thermal conductivity. The presence of rGO can enhance electrical conductivity due to the availability of more surface electrons from rGO. High surface areas, pore volumes, and thermal and electrical conductivity are significant factors for applications in the field of electrical and thermal engineering.

In addition, the effect of calcination conditions on the γ-Al_2_O_3_ and rGO hybrids was determined. As the calcination temperature increased, the crystallinity of the γ-Al_2_O_3_ and rGO hybrids was considerably enhanced as shown in their XRD spectra in the range of 500 K to 800 K [Fig. S3a†]. Previous reports confirmed this enhancement in the crystallinity of hybrids with calcination temperature.^23–25^ The content of rGO changes at different calcination temperatures and times. Thus, for the analysis, the calcination time was kept constant (1 h) and further, the calcination temperature was set as 500 K, 600 K, 650 K, 700 K, 750 K, and 800 K. For most of the samples, the characteristics peak for rGO is absent in the XRD spectra (Fig. S3(a)†). The peak for rGO usually appears at 24.50° in the hybrids. In the XRD spectra of the γ-Al_2_O_3_–rGO hybrids, the absence of the carbon peak may be due to the uniform mixture of rGO in alumina. The crystallinity of γ-alumina is major concern. Sharp, broad and prominent peaks are observed for γ-alumina at the higher calcination temperatures of 700 K, 750 K and 800 K. However, these peaks are weak or not prominent at the lower calcination temperatures of 500 K, 600 K, 650 K. In conclusion, an enhancement in the crystallinity of the γ-alumina occurs at higher calcination temperatures in the γ-Al_2_O_3_–rGO hybrids.

The plot of average nanorod diameter against calcination temperature is shown in Fig. S3b.† The TEM images of the same samples show average changes in the nanorod diameter as shown in Fig. S3(c–h).† The average diameter of the nanorods is enhanced from 8.89 nm to 20.72 nm as the calcination temperature changes from 600 K to 850 K. The average diameter of the nanorods is calculated to be 8.89, 10.23, 12.21, 18.21, 17.10 and 20.72 nm for the calcination temperature of 600, 650, 723, 750, 800 and 850 K, respectively. Further, statistical analysis of the changes in nanorods length due to the effect of the calcination conditions was conducted. Fig. S4(a–l)† show the TEM images of the γ-Al_2_O_3_ nanorods and rGO hybrids obtained at calcination temperatures of 723, 823, and 923 K with the calcination time ranging from 2 h to 5 h. Fig. 2e shows the statistical analysis of the γ-Al_2_O_3_ nanorods and rGO hybrids. All the TEM images shown in Fig. S4† confirmed that elongated and fine cross-linked γ-Al_2_O_3_–rGO nanorod hybrids were fabricated at temperatures above 723 K using this solvothermal method.

As the calcination temperature increased, the nanorod dimensions increased in both average length and diameter. This is evident from the statistical analysis, in which the average lengths of 286.11, 292.07 and 330.2 nm are found for the samples obtained at a calcination temperature of 723, 823 and 923 K with a 5 h processing time, respectively. The enhancement in calcination time resulted in an enhancement in length. By maintaining the calcination temperature at 723 K, the average lengths of 91.12, 121.23, 171.11, 241.49 and 286.12 nm were determined for the processing times of 1, 2, 3, 4 and 5 h, respectively. The higher calcination temperature and time resulted in more elongated nanorod structures in the alumina–rGO hybrids. Therefore, calcination temperature and time are the significant parameters to define the specific morphology, crystallinity, average lengths and diameters of nanorods.^24,25^

**Fig. 3:**
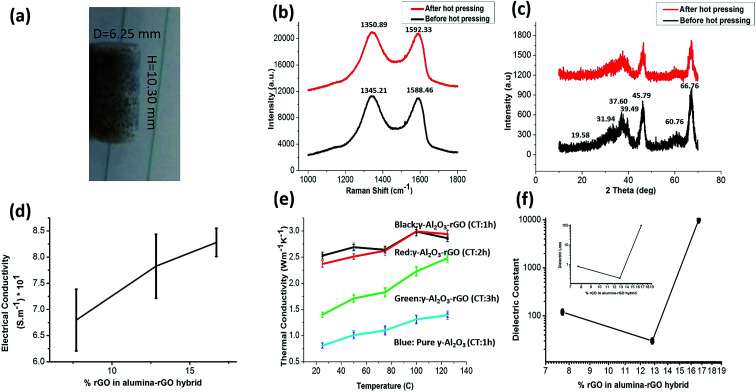
(a) Optical image of the hot pressed γ-Al_2_O_3_–rGO sample calcinated for 2 h. (b) Raman and (c) XRD spectra of γ-Al_2_O_3_–rGO (2 h calcination time) before and after hot pressing. (d) Electrical conductivity *vs.* % rGO with error bars. (e) Thermal conductivity as a function of temperature (black, red and green curves are for γ-Al_2_O_3_–rGO calcinated for 1, 2 and 3 h) with error bars. (f) Dielectric properties *vs.* % rGO in the γ-Al_2_O_3_–rGO hybrid with error bars.

The hot-pressed γ-Al_2_O_3_ and rGO nanohybrid samples were fabricated at a temperature of 900 °C. According to previous literature, under this condition, carbon and aluminum cannot react chemically.^20,26^ The samples prepared with the hot-pressing process were named 1 h, 2 h and 3 h-γ-Al_2_O_3_ and rGO hybrids and pure γ-Al_2_O_3_. The optical image of a hot-pressed sample of γ-Al_2_O_3_–rGO (2 h-calcination time) is shown in Fig. 3a. Hot pressing turns powder samples into solid cylinders, which are used in systematic studies of electrical, mechanical, dielectric, and thermal properties. Hot pressing can affect the quality of graphene.^27^ However, preserving the quality of graphene as much as possible is a major factor in the enhanced properties of graphene hybrids.^19–21^ The SEM morphology of all the samples after hot pressing is shown in Fig. S5(a–d).† It can be observed from the SEM images that most of the hot-pressed samples have a particle-like morphology with various particle sizes. The Raman and XRD spectra for γ-Al_2_O_3_–rGO calcinated for 2 h before and after hot pressing are shown in Fig. 3b and c, respectively. Before hot pressing, the D band for the sample is located at 1345.21 cm^−1^, while after hot pressing, it is located at 1350.89 cm^−1^. Before hot pressing, the G band is located at 1588.46 cm^−1^ and after hot pressing, it is located at 1592.33 cm^−1^. This shift in the D and G bands is due to the electronic interaction between γ-Al_2_O_3_ and rGO during the hot pressing process. The integrity of the alumina nanorods during hot pressing was not significantly affected, which was also confirmed by the XRD spectra before and after hot pressing as similar peaks for the same 2 h-calcinated samples were observed.

The hot-pressed γ-Al_2_O_3_–rGO hybrid samples calcinated for 1, 2, and 3 h and pure γ-Al_2_O_3_ (calcinated for 1 h) were chosen for electrical, mechanical, thermal and dielectric studies on γ-Al_2_O_3_ and the rGO hybrids with and without rGO. The hot-pressed samples were cut into squares for the four-probe measurements. Electrical conductivity as a function of the concentration of rGO in the hybrid is shown in Fig. 3d. It is also found that the electrical conductivity increases with an increase in rGO content. The electrical conductivities of γ-Al_2_O_3_–rGO calcinated for 1, 2, and 3 h were 8.2 × 10^1^ S m^−1^, 7.8 × 10^1^ S m^−1^, and 6.7 × 10^1^ S m^−1^, respectively. Slight conductivity (5.1 × 10^−10^ S m^−1^) is observed in the bare γ-Al_2_O_3_ samples, which confirms that bare alumina is highly nonconductive. Previous reports show that a low carbon content (2%) in alumina–carbon hybrids can significantly enhance conductivity (from 10^−12^ S m^−1^ to 10^−1^ S m^−1^).^28^ The improvement in the electrical properties is due to the heat treatment and it has been attributed to mechanisms such as restoration of the sp^2^ C–C bonds and cross-linking between reduced GO sheets during the thermal annealing process.^28^ Thus, calcination and hot press processing at higher temperatures are the reasons for the enhancement in the electrical conductivity of γ-Al_2_O_3_–rGO hybrids.^28,29^

The samples obtained *via* the solvothermal method show a significant increase and improvement in thermal conductivity, which is primarily because of the excess surface electrons and layered structure of rGO. Previously reported results indicated that thin rGO layers independently have high conductivities.^29,30^ The thermal conductivity of pure alumina prepared *via* hydrolysis and a solvothermal method was reported to be 0.5 W m^−1^ K^−1^ at 75 °C.^20^ The thermal conductivity of γ-Al_2_O_3_–rGO with 1, 2 and 3 h-calcination times and bare γ-Al_2_O_3_ (1 h-calcination time) as a function of temperature is shown in Fig. 3e. At room temperature (25 °C), the thermal conductivities of pure γ-Al_2_O_3_ and γ-Al_2_O_3_–rGO (3, 2 and 1 h calcination times) were found to be 0.81, 1.4, 2.37, and 2.53 W m^−1^ K^−1^, respectively. As the temperature increased, the thermal conductivity gradually increased in all the γ-Al_2_O_3_–rGO hybrids and bare γ-Al_2_O_3_. Because of the lack of free electrons, nonmetallic ceramic materials are usually thermal insulators.^20^ In the case of ceramic materials, porosity is one of the main reasons for a decrease in the overall thermal conductivity. The thermal conductivity of γ-Al_2_O_3_–rGO is enhanced by more than 80% compared to that of bare γ-Al_2_O_3_ when there is an increase in rGO content in the γ-Al_2_O_3_–rGO hybrid with a 3 h-calcination time.

The dielectric properties of the γ-Al_2_O_3_–rGO hybrids and bare γ-Al_2_O_3_ were measured using an LCR meter as shown in Fig. 3f. The dielectric properties of the hybrids were measured at a frequency of 1 kHz. For γ-Al_2_O_3_, its dielectric constant is found to be around 9.8, which is closer to that reported previously for pure alumina.^31,32^ For the 3 h-calcinated hybrid, the dielectric constant significantly increased by a factor of 12, which indicates the presence and proximity of a first percolation threshold. However, when the rGO content is enhanced in the hybrids by decreasing calcination time to 2 h, the dielectric constant further decreases and approaches the value of pure γ-Al_2_O_3_. This is attributed to an anomalous trend that produces drastic changes in most ceramic materials and matrix microstructures. By further decreasing the calcination time of the hybrids to 1 h, the dielectric constant increased by four orders of magnitude, indicating the presence of a second percolation threshold, which is achieved through this higher value of dielectric constant.^33,34^ Similarly, the dielectric loss indicates very similar behavior in the real part of the dielectric constant as shown in the inset of Fig. 2f. The dielectric loss in the Al_2_O_3_–rGO hybrid with a 1 h-calcination time increased significantly since this hybrid contained more rGO, which could make conductive layer-networks of rGO in between the alumina nanorods. This would cause significant leakage current and thus result in high dielectric loss. The existence of a double percolation threshold in γ-Al_2_O_3_ and the rGO hybrids can be significant for technological and practical applications because it can be used to enhance the dielectric properties in γ-Al_2_O_3_ and rGO hybrids with the addition of a small percentage of rGO in the hybrids. Previous reports have shown that a small amount of rGO in hybrids can enhance dielectric properties to a great extent.^35^

**Fig. 4:**
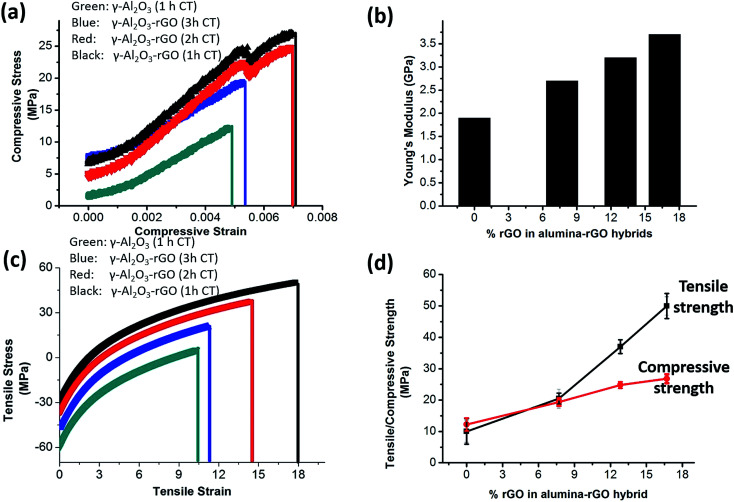
(a) Compressive stress strain curves for the γ-Al_2_O_3_–rGO hybrids calcinated for 1, 2 and 3 h, (b) compressive Young's modulus as a function of % rGO, (c) tensile stress/strain curves for the γ-Al_2_O_3_–rGO hybrids calcinated for 1, 2 and 3 h and (d) compressive and tensile strength as a function of % rGO for the γ-Al_2_O_3_–rGO hybrids with error bars.

From the compressive and tensile stress strain curves shown in Fig. 4(a) and (c), it is evident that with an increase in rGO content in the hybrid, the mechanical compressive and tensile strength increase as compared to pure alumina. This further caused increased strength in the alumina hybrids, *i.e.*, higher compressive, tensile strength and higher compressive Young's modulus values were obtained [Fig. 4(b) and (d)]. The enhanced mechanical properties of the γ-Al_2_O_3_ and rGO hybrids can be attributed to covalent interaction of rGO with γ-Al_2_O_3_ and efficient load transfer between rGO and the γ-Al_2_O_3_ nanorods.^19,30^ Furthermore, this is closely associated with the elongated and fine γ-Al_2_O_3_ nanorods and atomic-level rGO layers having covalent interactions with γ-Al_2_O_3_.^15,30,36^ The previously reported Young's modulus values for pure alumina are much higher and the tensile strength was reported to be between 0.5 and 1 GPa.^37–39^ The Young's modulus of γ-Al_2_O_3_–rGO calcinated for 1, 2 and 3 h and γ-Al_2_O_3_ calcinated for 1 h is calculated to be 3.7, 3.2, 2.65 and 1.80 GPa, respectively. In our case, the lower tensile and compressive strength in alumina may be due to the presence of powder instead of single crystals of alumina. The increase in calcination temperature reduced the wt% of rGO in hybrid. Thus, the hybrids with a lower calcination temperature have more strength. The maximum value of Young's modulus (3.7 GPa) was obtained for the 1 h-calcinated alumina–rGO hybrid. Fig. S6† presents the Young's modulus values as a function of the aspect ratio of the nanorods for these samples. With an increase in the aspect ratio of the nanorods, the Young's modulus increases. The maximum aspect ratio was calculated for the sample calcinated for 1 h. Thus, the elongated dimensions of the nanorods are the major cause of their high mechanical strength. In the γ-Al_2_O_3_–rGO monoliths, a higher calcination temperature enhanced the length, diameter and aspect ratios of the γ-Al_2_O_3_ nanorods. The presence of more rGO and elongated alumina rods with higher aspect ratio determine the interface interaction between the rGO platelets and alumina. RGO, which is obtained by the thermal treatment at high temperature, exhibits a high surface area, which is beneficial for higher mechanical and interfacial interaction or practical adhesion between the rGO platelets and higher aspect ratio elongated structures of alumina in the hybrids. Consequently, higher mechanical strength is observed in the hybrids. A 90% increase in tensile strength and 75% increase in compressive strength occur when the content of rGO increased from 0 to 7.705 wt% in the hybrid calcinated for 3 h. With an increase in the rGO content, the alumina–rGO hybrids show higher Young's modulus values. A comparison of the properties of these hybrids with previously reported studies on alumina–rGO is shown in Table 1.

**Table 1:** Comparison of the properties of the γ-Al_2_O_3_–rGO hybrids (obtained *via* solvothermal and hot press processing) with that in previous reports on alumina–graphene hybrids

Method of fabrication	BET surface area (m^2^ g^−1^)	Bulk density (g cm^−3^)	Thermal conductivity (W m^−1^ k^−1^) at 25 °C	Conductivity (S m^−1^)	Compressive strength (MPa)	Young's modulus (GPa)	Tensile strength (MPa)	Dielectric properties	
								D.C	D.L
γ-Al_2_O_3_–rGO-1 CT (this work)	379	0.92	2.53	8.2 × 10^1^	26.79	3.7	49.98	10^4^	10^2^
γ-Al_2_O_3_–rGO-2 CT (this work)	408	1.37	2.37	7.8 × 10^1^	24.80	3.2	37.05	30	0.2
γ-Al_2_O_3_–rGO-3 CT (this work)	361	1.61	1.4	6.7 × 10^1^	19.41	2.7	20.47	120	0.8
Alumina-30% rGO/PEI^20^	327	1.65	1.3	—	—	—	—	—	—
40 wt% rGO–Al_2_O_3_ cores shell flakes^17^	119.71	0.003	—	—	—	—	—	—	—
Al_2_O_3_–rGO microwave sintering^21^		92.2% (relative density)	—	0.39	—	180	—	—	—
G-alumina (30%)/epoxy^40^	—	—	0.329	—	—	—	57.34	—	—

To the best of our knowledge, the as-fabricated γ-Al_2_O_3_ and rGO hybrids obtained *via* a solvothermal method show a significant improvement in electrical, mechanical, thermal, dielectric, and physical properties. The hybrid calcinated for 1 h shows good enhancement in its electrical conductivity (8.2 × 10^1^ S m^−1^) due to the availability of more surface electrons from rGO. This is best value reported to date for conductivity in an alumina–rGO hybrid as compared to previously reported maximum value.^21^ After the hot pressing process, there was a significant increase in the electrical conductivity values in the hybrids when the calcination time decreased from 3 h to 1 h. Further, the thermal conductivity of γ-Al_2_O_3_–rGO is enhanced by more than 80% compared to that of bare γ-Al_2_O_3_ when there is an increase in rGO content up to 7.705 wt% in the γ-Al_2_O_3_ and rGO hybrids. In comparison with previous reports, there is a 77% increase in thermal conductivity on using this solvothermal method to obtain γ-Al_2_O_3_ and rGO hybrids.^20,40^

In hybrids, higher rGO content can enhance their physical properties, such as electrical, thermal, dielectric and mechanical properties, to a great extent. Furthermore, the high surface area, more interfacial interaction of rGO, and higher rGO platelets can significantly enhance the physical properties of the hybrids. Physical properties such as the BET surface area and bulk density are also improved as shown in Table S1.† In addition, the elongated dimensions of the nanorods are the major cause for the higher mechanical strength in these hybrids. A 90% increase in tensile strength and 75% increase in compressive strength occur when the content of rGO is increased up to 7.707% in the hybrid. The dielectric constant increases by four orders of magnitude through a second percolation threshold with the addition of a small amount of rGO in the hybrid. The enhancement in physical properties is due to the well-aligned, elongated and fine nanorod morphology of alumina in the hybrids and calcination and hot press processing further played important roles by sustaining the quality of rGO in the hybrids.

In conclusion, hybrids composed of rGO and alumina monoliths were prepared *via* the simple impregnation of GO in cyclohexane and aluminum isopropoxide by a solvothermal reaction. An increase in calcination temperature and time resulted in enhanced crystallinity in the γ-Al_2_O_3_ nanorods and rGO hybrids. This caused further enhancement in the diameters and lengths of the nanorods in the hybrid structures. After calcination and hot-press processing, Al_2_O_3_–rGO monoliths were obtained with enhanced electrical, mechanical, thermal, and physical properties. This study shows best reported electrical conductivity (8.2 × 10^1^ S m^−1^), higher dielectric constant by four orders of magnitude and 77% increase in thermal conductivity (1.4 W m^−1^ K^−1^) in comparison with previous reports. Hot pressing at 900 °C ensured the complete reduction of GO and the crystallinity of γ-Al_2_O_3_, resulting in enhanced physical properties in the hybrids. The elongated and fine γ-Al_2_O_3_ nanorod morphology, atomic-level layered structure and excess surface free electrons of rGO resulted in the best reported BET surface area (408 m^2^ g^−1^ in the 2 h-calcinated alumina–rGO hybrid), best thermal conductivity (2.53 W m^−1^ K^−1^ in the 1 h-calcinated alumina–rGO hybrid), and relatively small density (0.92 g cm^−3^ in the 1 h-calcinated alumina–rGO hybrid) and high strength (3.7 GPa in the 1 h-calcinated alumina–rGO hybrid). Thus, these nanohybrids of alumina monoliths and rGO can be further applied as catalysts, electrolytes, and as electrochemically active materials because of their nanometer dimensions and enhanced physical properties.

## Conflicts of interest

The authors declare no competing financial interests.

## Supplementary Material

RA-008-C8RA00095F-s001
