# Supplementary material for: Enhanced physical properties of γ-Al2O3–rGO hybrids prepared by solvothermal and hot-press processing
Source: RSC Adv. 2018 Feb 22;8(15):8329–37. doi: 10.1039/c8ra00095f (PMC9078560; doi:10.1039/c8ra00095f)
Supplement: RA-008-C8RA00095F-s001 [file RA-008-C8RA00095F-s001.pdf]

Electronic Supplementary Material (ESI) for RSC Advances.
This journal is © The Royal Society of Chemistry 2018

Enhanced physical properties of γ-Al_2O_3-rGO hybrids prepared by solvothermal and hot-press processing

Mujtaba Ikram[1]*, Zhuchen Tao[1], Jianglin Ye[1], Hafiz Adil Qayyum[2], Xuemei Sun [1] and Jin Xu[1]

[1]Key Laboratory of Materials for Energy Conversion, Chinese Academy of Sciences, & Department of Materials Science and Engineering, University of Science and Technology of China, Hefei, Anhui 230026, P. R. China

[2]Physics Department, King Fahd University of Petroleum and Minerals, Dhahran 31261, Saudi Arabia

*Corresponding author: mujtaba@mail.ustc.edu.cn

Supporting Information

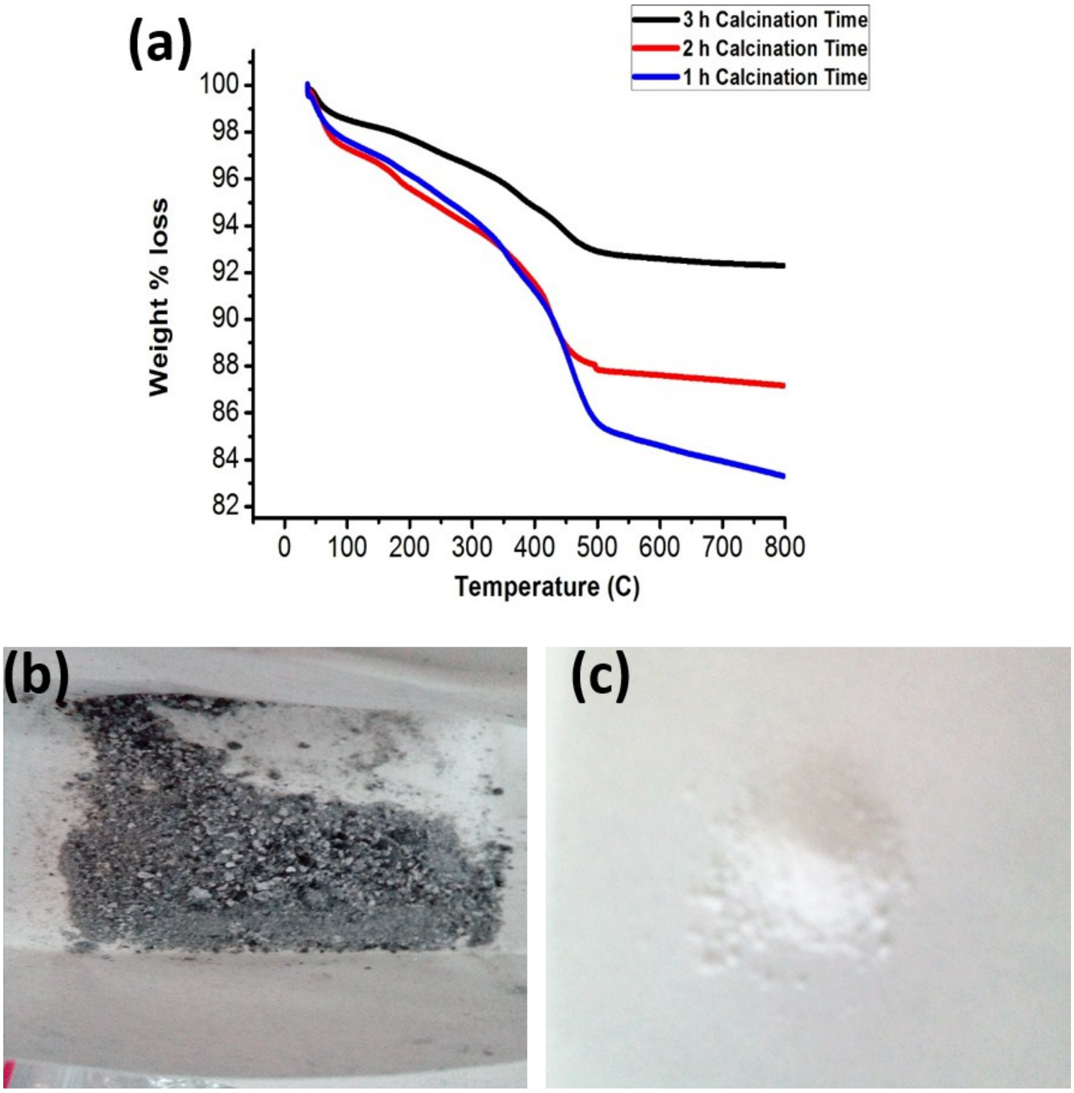

Fig.S1 (a) TGA curves of γ-Al_2O_3-rGO hybrids using calcination time of 1-h, 2-h and 3-h in air atmosphere up to 800°C. Powder colors of $Al(O)_x$/GO (b) before (c) after calcination at 723 K for 2 h

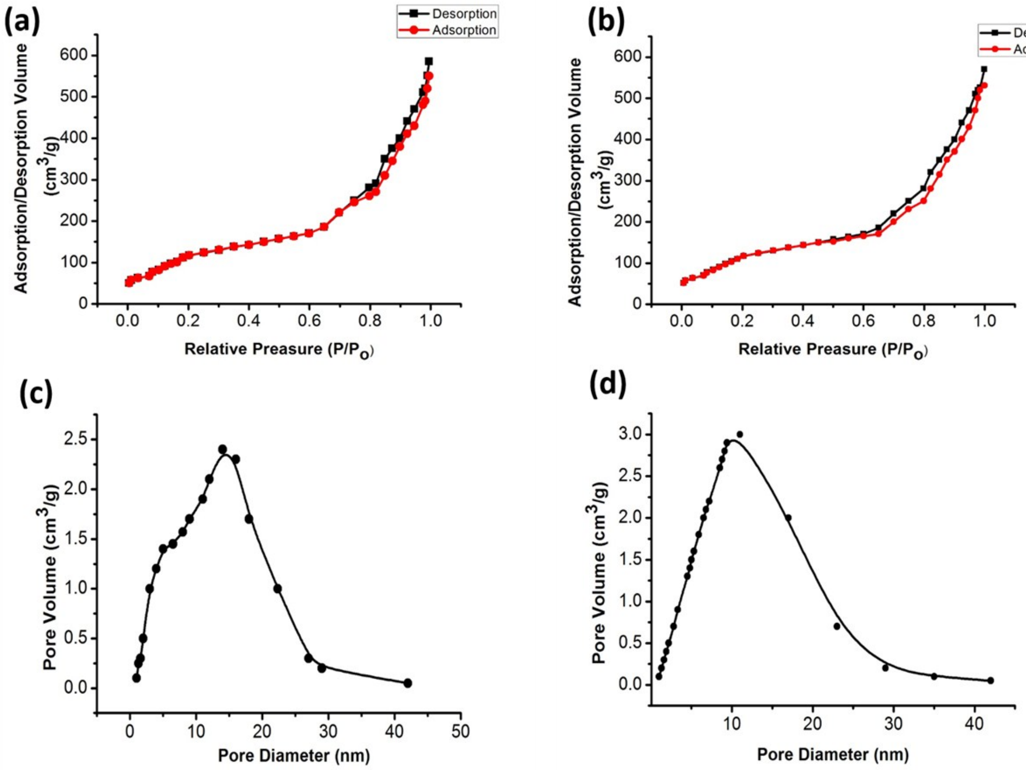

Fig. S2 (a, c) N_2 adsorption-desorption and pore size distribution curves for pure γ-Al_2O_3 (1-h calcination time) and (b, d) for γ-Al_2O_3-rGO hybrid (1-h calcination time)

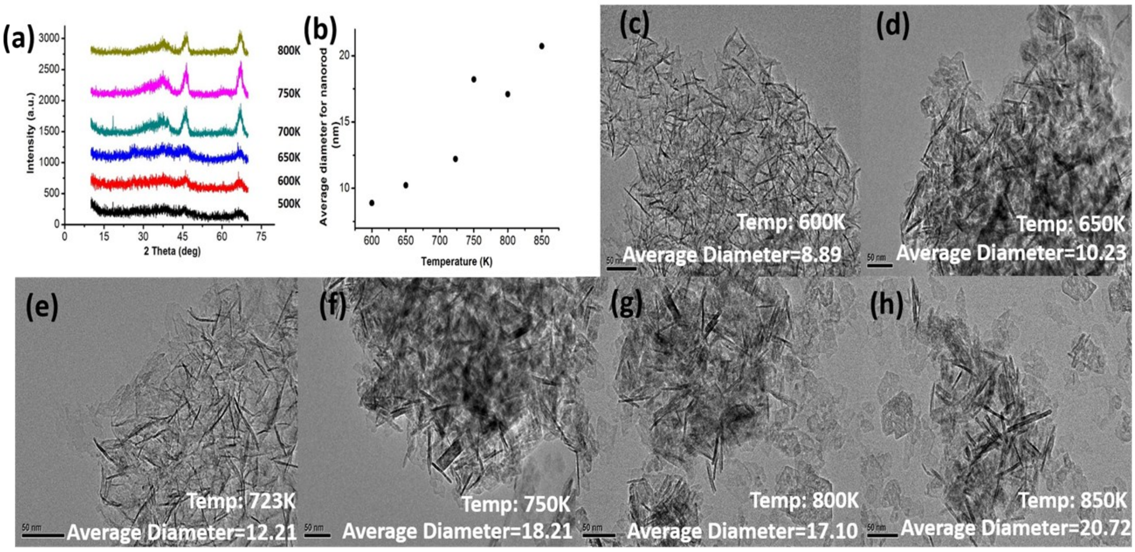

Fig.S3 (a) XRD of γ-Al_2O_3-rGO hybrids taken from 500 K to 800 K (b) Average diameter of nano rods (nm) as function of calcination temperature (K) (c-h) TEM images showing variations in diameters of nano rod structures with various calcination temperatures. Units for diameters are in (nm)

Sample Type	BET surface area [$m^2 g^{-1}$]				Bulk density [g/cm^3]			
Our Method (Solvothermal)	γ-Al_2O_3 (1h C.T)	γ-Al_2O_3-rGO (3h Calcination Time)	γ-Al_2O_3-rGO (2h Calcination Time)	γ-Al_2O_3-rGO (1h Calcination Time)	γ-Al_2O_3 (1h C.T)	γ-Al_2O_3-rGO (3h Calcination Time)	γ-Al_2O_3-rGO (2h Calcination Time)	γ-Al_2O_3-rGO (1h Calcination Time)
	280	361	408	379	2.75	1.61	1.37	0.92
Meso-porous Al_2O_3-rGO	Al_2O_3		Al_2O_3-rGO		Al_2O_3		Al_2O_3-rGO	
	243		327		2.40		1.65	
Core-shell flakes Al_2O_3-rGO	Al_2O_3		Al_2O_3-rGO		Al_2O_3		Al_2O_3-rGO	
	286.62		119.71		2.816		0.003	
In situ deposition Al_2O_3-rGO	Al_2O_3		Al_2O_3-rGO		Al_2O_3		Al_2O_3-rGO	
	N/A		242.4		N/A		N/A	

Table S1. BET surface area and density comparison for γ-Al_2O_3-rGO (1, 2 and 3 h calcination time) and pure γ-Al_2O_3 (1 h calcination time) with previous reports

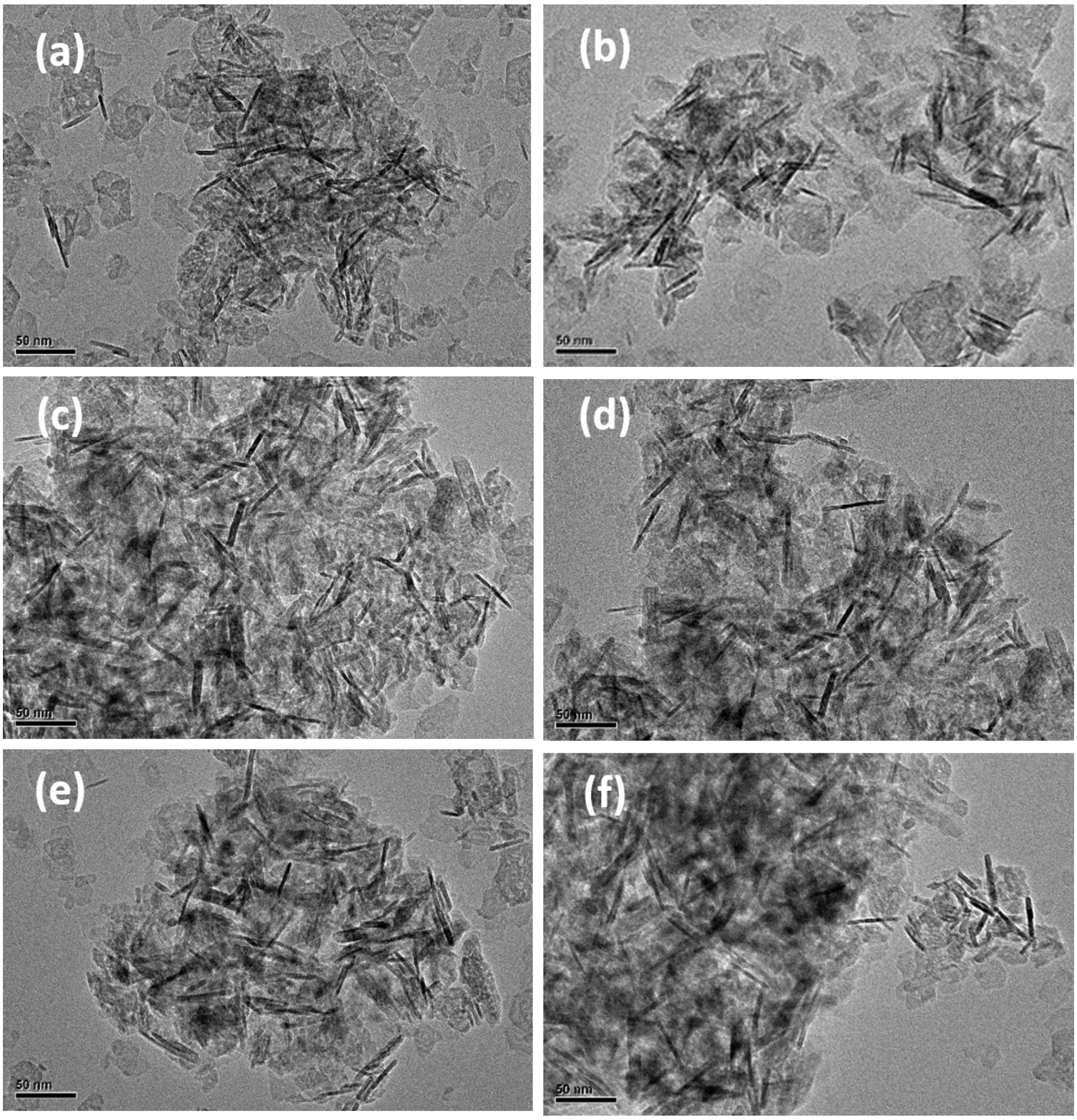
(a)
50 nm
(b)
50 nm
(c)
50 nm
(d)
50 nm
(e)
50 nm
(f)
50 nm

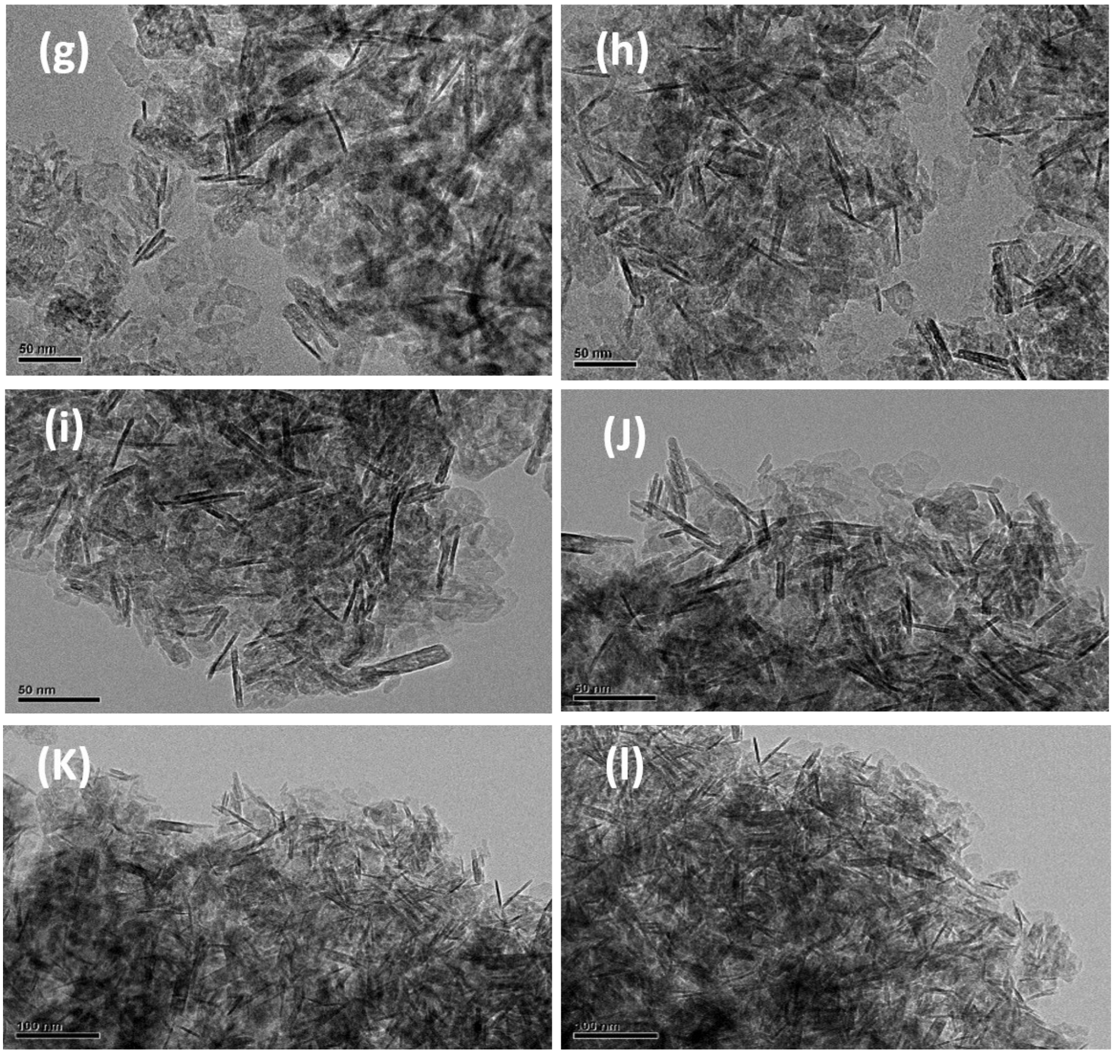

Fig. S4 TEM morphology of γ-Al_2O_3-rGO nano rods using different calcination time and calcination temp (a-d) 2,3,4 and 5 h at 723 K (e-h) 2,3, 4 and 5 h at 823 K (i-l) 2,3,4 and 5 h at 923 K

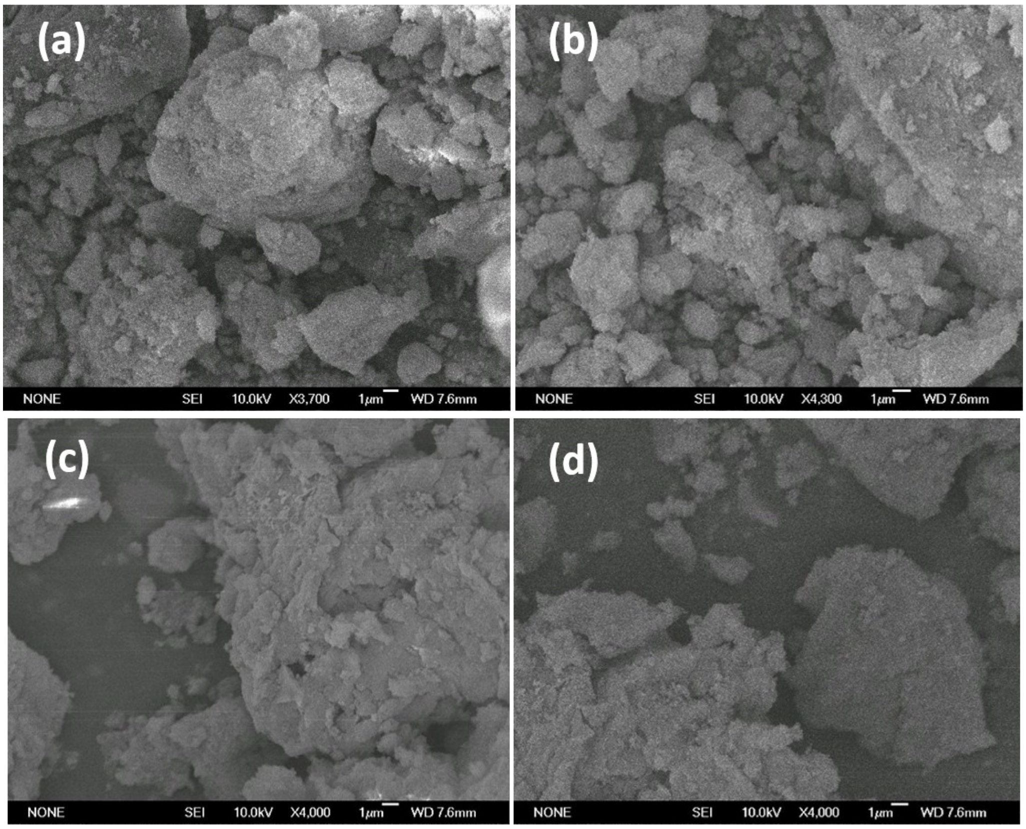

Fig. S5 SEM images of hot pressed samples (a) γ-Al_2O_3-rGO (1 h calcination time) (b) γ-Al_2O_3-rGO (2 h calcination time) (c) γ-Al_2O_3-rGO (3 h calcination time) and (d) pure γ-Al_2O_3 (1 h calcination time)

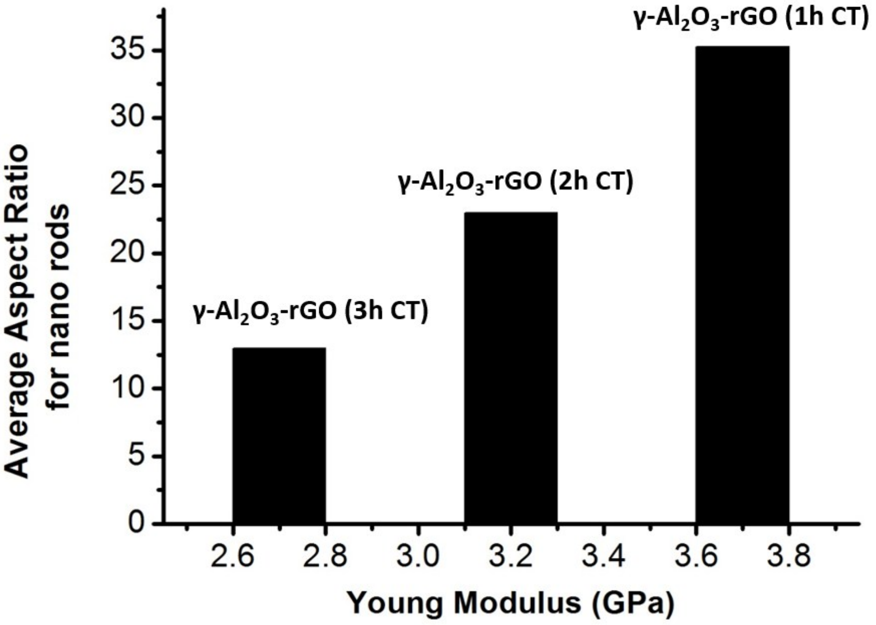

Fig. S6 Young Modulus as function of average aspect ratio of nano rods in hot pressed samples γ-Al_2O_3-rGO hybrids with 1, 2 and 3 h calcination time

References

1. A. M. Jastrzębska, J. Karcz, R. Letmanowski, D. Zabost, E. Ciecierska, J. Zdunek, E. Karwowska, M. Siekierski, A. Olszyna and A. Kunicki, *Appl. Surf. Sci.*, 2016, **362**, 577–594.
2. K. Bhowmik, A. Chakravarty, S. Bysakh and G. De, *Energy Technol.*, 2016, **4**, 1409–1419.
3. A. M. Jastrzębska, A. R. Olszyna, J. Jureczko and A. Kunicki, *Int. J. Appl. Ceram. Technol.*, 2015, **12**, 522–528.
